# Role of IL-37- and IL-37-Treated Dendritic Cells in Acute Coronary Syndrome

**DOI:** 10.1155/2021/6454177

**Published:** 2021-08-21

**Authors:** Ruirui Zhu, Fangyuan Zhang, Chengliang Pan, Kunwu Yu, Yucheng Zhong, Qiutang Zeng

**Affiliations:** ^1^Department of Cardiology, Union Hospital, Tongji Medical College, Huazhong University of Science and Technology, Wuhan 430022, China; ^2^Department of Dermatology, Union Hospital, Tongji Medical College, Huazhong University of Science and Technology, Wuhan 430022, China

## Abstract

As a chronic inflammatory disease, atherosclerosis is a leading cause of morbidity and mortality in most countries. Inflammation is responsible for plaque instability and the subsequent onset of acute coronary syndrome (ACS), which is one of the leading causes of hospitalization. Therefore, exploring the potential mechanism underlying ACS is of considerable concern, and searching for alternative therapeutic targets is very urgent. Interleukin-37 (IL-37) inhibits the production of proinflammatory chemokines and cytokines and acts as a natural inhibitor of innate and adaptive immunity. Interestingly, our previous study with murine models showed that IL-37 alleviated cardiac remodeling and myocardial ischemia/reperfusion injury. Of note, our clinical study revealed that IL-37 is elevated and plays a beneficial role in patients with ACS. Moreover, dendritic cells (DCs) orchestrate both immunity and tolerance, and tolerogenic DCs (tDCs) are characterized by more secretion of immunosuppressive cytokines. As expected, IL-37-treated DCs are tolerogenic. Hence, we speculate that IL-37- or IL-37-treated DCs is a novel therapeutic possibility for ACS, and the precise mechanism of IL-37 requires further study.

## 1. Introduction

Atherosclerosis is a chronic inflammatory disease and the basis of acute coronary syndrome (ACS), including acute myocardial infarction and unstable angina [1]. ACS is a result of coronary plaque erosion or rupture, where inflammation plays a central role [1–3]. Inflammatory reactions are triggered by the discharge of intracellular substances from necrotic myocardium after MI, followed by activation of nuclear factor- (NF-) *κ*B, which is an essential transcription factor that controls the expression of inflammatory genes, such as chemokines and cytokines. After that, several inflammatory cells, including macrophages, neutrophils, and T cells, are recruited into the infarcted heart. Moreover, one famous clinical study has revealed that tumor necrosis factor (TNF) and IL-6 levels are positively correlated with the ACS severity and in-hospital mortality [4]. Recently, several clinical experiments have demonstrated that high-sensitivity C-reactive protein (hs-CRP) is a biomarker of inflammation and atheromatous plaque vulnerability [5–7]. In addition, a previous publication showed that a poor clinical outcome after myocardial infarction (MI) can be predicted by increased serum CRP concentration [8]. In our study, we also showed that hs-CRP levels were significantly higher in patients with ACS [9]. Strategies that target inflammation aim for timely resolution of inflammation without interfering with the healing responses in MI.

## 2. IL-37 and Dendritic Cells

Interleukin-37 (IL-37) was initially discovered in 2000 as a natural inhibitor of innate and adaptive immunity [10, 11]. Although it is rarely expressed physiologically and in low amounts, IL-37 expression can be stimulated by proinflammatory cytokines or toll-like receptor ligands, which, in turn, inhibit proinflammatory cytokines [10, 12, 13]. As ligand for the orphan receptor IL-1R8, IL-37 functions extracellularly to decrease innate inflammation after lipopolysaccharide (LPS) activation [14]. Meanwhile, IL-37 translocates to the nucleus and inhibits proinflammatory cytokines [15]. In addition, transgenic IL-37 mice are protected from several disease models, including LPS-induced septic shock [11], dextran sulfate-induced colitis [16], concanavalin A-induced hepatitis [17], and cerebral ischemia/reperfusion injury [18]. Arterial calcification is a predictor of coronary heart events. Moreover, IL-37 is highly expressed in human atherosclerotic plaque foam cells [19] and elevated in patients with arterial calcification [20], indicating that IL-37 may be associate with the progression of coronary heart diseases.

Dendritic cells (DCs), one of the antigen-presenting cells, orchestrate both immunity and tolerance; therefore, they are pivotal targets for immunotherapy [21–25]. One previous publication showed that semimature DCs are tolerogenic and mature DCs are immunogenic [26]. Unlike mature DCs, tDCs are associated with an increased secretion of immunosuppressive cytokines, decreased IL-12, and reduced costimulatory molecules and MHC-II and eventually induce Treg cells and deliver inadequate signals for effector T cell activation [24, 25, 27]. Several clinical studies have used autologous tolerogenic dendritic cells (tDCs) for therapy in auto-immune diseases [28–30]. Of note, we have demonstrated that IL-37-treated DCs are tolerogenic. Therefore, these autologous tDCs are a novel therapeutic possibility for ACS.

## 3. Role of IL-37 in ACS

Atherosclerosis is a chronic inflammatory disease characterized by atherosclerotic plaque formation and hardening of the arterial wall [31]. Inflammation plays a central role in the development of ACS [1–3]. IL-37 is a suppressor of innate and adaptive immune reactions [11, 32]. Moreover, there are several similar observations that IL-37 restricted the local inflammatory responses by inhibiting recruitment of inflammatory cells in colitis, liver inflammatory injury, and psoriasis [16, 33, 34]. Interestingly, IL-37 alleviated cardiac function via inhibition of myocardial inflammation in old endotoxemic mice [35]. Hence, IL-37 may be associated with the pathophysiology of ACS. Indeed, several laboratories have investigated the effects of IL-37 in ACS [36–38]. IL-37 levels were found to be elevated and negatively correlated with the left ventricular ejection fraction in patients with ACS in a previous study [39]. Importantly, we also demonstrated that IL-37 elevation is systemic rather than local [39]. To the best of our knowledge, the positive association between IL-37 and inflammatory biomarkers may be due to the inflammatory cytokines that stimulate IL-37 expression [40]. Furthermore, we and other groups previously reported that plasma IL-37 levels were increased in patients with ACS and predicted a worse clinical outcome after ST-segment elevation acute MI [9, 36]. In addition, IL-37 suppressed neutrophil recruitment in vivo and migration ability in vitro in ischemia/reperfusion models [41]. We observed that IL-37 treatment alleviated ventricular remodeling after MI by inhibiting the infiltration of several inflammatory cells (Figure S1) [42]. Of note, we also demonstrated that the inflammatory reactions inhibited by IL-37 were independent of the infarct size after MI by ruling out the influence of the secondary immune response due to the smaller infarct size [42]. Together, the above findings reveal that IL-37 plays a protective role in ACS.

NF-*κ*B signaling is activated and involved in myocardial remodeling after acute myocardial infarction (AMI), and blocking this pathway improves the cardiac function and survival in mouse and rat models [38, 41]. Our group and other laboratories have demonstrated that inflammatory NF-*κ*B signaling was effectively inhibited after treatment with IL-37, respectively [38, 41]. Moreover, IL-37 can bind to the IL-18 receptor, and left ventricular function can be improved by blocking IL-18 [43, 44]. Importantly, our previous publication revealed that IL-37, IL-18, and IL-18 binding protein (IL-18BP) levels were increased in patients with ACS [39]. However, the affinity of IL-18BP for IL-37 is far lower than that of IL-18 [45]. Of note, IL-37 has not been shown to be a receptor antagonist for IL-18 because IL-37 did not antagonize the activities of IL-18, though IL-37 binds to IL-18 receptor *α*-chain (IL-18R*α*) [12]. Therefore, the inhibitory effect of IL-37 on IL-18 is rather complex. Previous studies have reported that the anti-inflammatory role of IL-37 requires the receptors IL-18R*α* and IL-1R8 [46, 47]. Therefore, IL-18R*α* and IL-1R8 knockout mice are required to confirm this interaction in ACS.

## 4. Role of IL-37 in Apoptosis and Myocardial Fibrosis

Apoptosis is responsible for the loss of surviving cardiomyocytes in the late stage of MI after the early ischemic death of cardiomyocytes [48]. Apoptosis was associated with cardiomyocyte death within 4 h and lasted for up to 30 days after coronary artery ligation [49, 50]. IL-37 ameliorated cardiomyocyte apoptosis after MI, and the antiapoptotic effects on cardiomyocytes resulted in a smaller infarct size on days 1 and 28 [42]. Importantly, the Bax/Bcl-2 ratio is a critical factor for cell apoptosis [51]. As expected, we found that the Bax/Bcl-2 ratio was restored in IL-37–treated mice and cardiomyocytes, revealing that IL-37 inhibited cardiomyocyte apoptosis by decreasing the Bax/Bcl-2 ratio [42]. Moreover, smooth muscle cell apoptosis significantly contributes to plaque instability [52]. Cardiac fibrosis is a central tenet of post-MI ventricular remodeling. A previous study showed that IL-37 reduced collagen degradation and inhibited smooth muscle cell apoptosis, which in turn increased plaque stability [53]. In line with this publication, we also discovered that the protein levels of matrix metalloproteinase-2 were significantly lower in IL-37–treated mice than in controls and that IL-37–treated mice exhibited markedly reduced fibrotic areas in both the infarct and remote areas [42]. Hence, IL-37 ameliorated myocardial fibrosis in infarcted hearts.

## 5. Effect of IL-37 on T Cells and Cytokines

Based on the diverse cytokines they produce, CD4+ T cells can be divided into different profiles. CD4+ T cells include Th1, Th2, Th17, and regulatory T cells (Tregs). Previous studies have demonstrated that the Th1 response contributes to plaque rupture, and inhibition of the Th1 response alleviates atherogenesis [54, 55]. In contrast, the Th2 response contributes to the anti-inflammatory response and promotes collagen deposition, which facilitates wound healing and fibrosis [56–58]. Moreover, the Th1/Th2 imbalance may lead to the rupture of plaque and result in the occurrence of ACS [59]. Strikingly, the Th1/Th2 ratio in peripheral T cells is decreased after statin treatment in patients with MI [60, 61]. The above studies indicate that the Th1/Th2 balance may affect reparative fibrosis. Of note, Tregs/Th17 imbalance has been observed both in patients with ACS and in ApoE-/- mice from a C57BL/6 background in our laboratory [62, 63]. Moreover, two previous publications elucidated that Th1/Th2 imbalance promoted, while regulatory T cells alleviated cardiac remodeling after MI [59, 64]. Taken together, these findings demonstrate that plaque destabilization and the onset of ACS are associated with distinct CD4+ T cell subpopulations. More importantly, we also demonstrated that Treg cells were upregulated, but Th1 and Th17 cells were suppressed by IL-37 treatment both in ACS patients [9] and ApoE-/- mice [65]. Hence, we surmise that the protective role of IL-37 in patients with ACS may be due to its different effects on Treg cells, Th1 cells, and Th17 cells.

Additionally, different cytokines play a complex role in ACS. Interferon*-γ* (IFN-*γ*) is a proinflammatory cytokine that is highly expressed in atherosclerotic lesions. At the same time, higher IL-17- and IL-17-induced cytokines have been detected in patients with ACS [66, 67]. Meanwhile, overexpression of IL-10 or IL-10 deficiency demonstrated remarkable amelioration or exacerbation of the development of atherosclerosis [68, 69]. Moreover, proinflammatory TNF was shown to be associated with MI pathogenesis, and cardiomyocytes were found to be a vital source of TNF production [70, 71]. Importantly, our laboratory has demonstrated that IL-17A contributes to ventricular remodeling in both MI and ischemia/reperfusion models [72, 73]. We also found that IFN-*γ* and IL-17 levels were significantly higher in patients with ACS [9]. Interestingly, we also demonstrated that IL-37 treatment inhibited IFN-*γ* and IL-17 mRNA expression and increased IL-10 and TGF-*β* mRNA levels in activated peripheral blood mononuclear cells [9]. Notably, in a mouse myocardial ischemia/reperfusion injury and MI model, we discovered that proinflammatory IL-6, IL-1*β*, and TNF-*α* were remarkably inhibited by IL-37 treatment, while IL-10, an anti-inflammatory cytokine, was significantly upregulated by IL-37 [41, 42]. In addition, western blotting analysis further confirmed that the protein levels of these cytokines were similar to the mRNA levels [42].

IL-10 is a classic anti-inflammatory cytokine. Several previous studies have explored the mechanism of IL-10 in post-MI ventricular remodeling [74–76]. Although exogenous administration of IL-10 plays a protective role, the endogenous absence of this cytokine is not sufficient to influence ventricular remodeling after MI [74–76]. Indeed, IL-10 remarkably inhibited the infiltration of inflammatory cells and expression of inflammatory cytokines in the myocardium [76]. Moreover, upregulation of IL-10 was observed in infarcted hearts after Treg cells transfer, and conventional T cells were converted into IL-10-producing Treg cells by Treg cells [64, 77]. Interestingly, our previous findings revealed that IL-10 was upregulated after treatment with IL-37 or adoptive transfer with tDCs [42]. One possible interpretation is that direct secretion by tDCs and/or expansion of Treg cells is responsible for the upregulation of IL-10. In fact, the ability of Treg cells to suppress pathogenic Th17 cell responses was endowed by IL-10 [78]. Of note, our clinical study confirmed that IL-37 increased anti-inflammatory IL-10, which plays a protective role in patients with ACS [9]. Consequently, our results indicate that the therapeutic effect of IL-37 in ACS is mediated by two mechanisms: decreased proinflammatory IFN-*γ* and IL-17 and increased anti-inflammatory IL-10. Unexpectedly, nanomolar concentrations of IL-37 were identified to cause excessive inflammation in activated peripheral blood mononuclear cells [14]. The observation offers another diverse possibility and therefore the precise regulation of IL-37 in ACS needs further study. Indeed, concentration-dependent effects of IL-37 have already been demonstrated [79]. Interestingly, high plasma IL-37 levels indicated poor prognoses in ACS [37]. Similar to B-type natriuretic peptide in heart failure [80], endogenous production of increased IL-37 may not be sufficient to counterbalance the proinflammatory and anti-inflammatory cytokine levels, and therefore, may be unable to attenuate inflammation in patients.

## 6. Therapeutic Potential of IL-37-Treated Tolerogenic Dendritic Cells in ACS

Although the priming of antigen-specific immune responses after tissue injury or microbial infection has been well studied, the function of DCs in immunologic tolerance is just beginning to be investigated. Autoimmunity to cardiac myosin [81] or TnI [82] is associated with poor clinical outcomes after MI because it promotes secondary myocardial injury. We and other researchers have elucidated that thymic stromal lymphopoietin–conditioned DCs induce Treg differentiation and eventually ameliorate atherosclerosis development in ApoE/mice in addition to protecting nonobese diabetic mice against diabetes [83, 84]. Another report indicated that myosin-primed tDCs attenuate autoimmune myocarditis [85]. The above studies together indicated that tDCs play a beneficial role in these autoimmune disease models.

**Figure 1:**
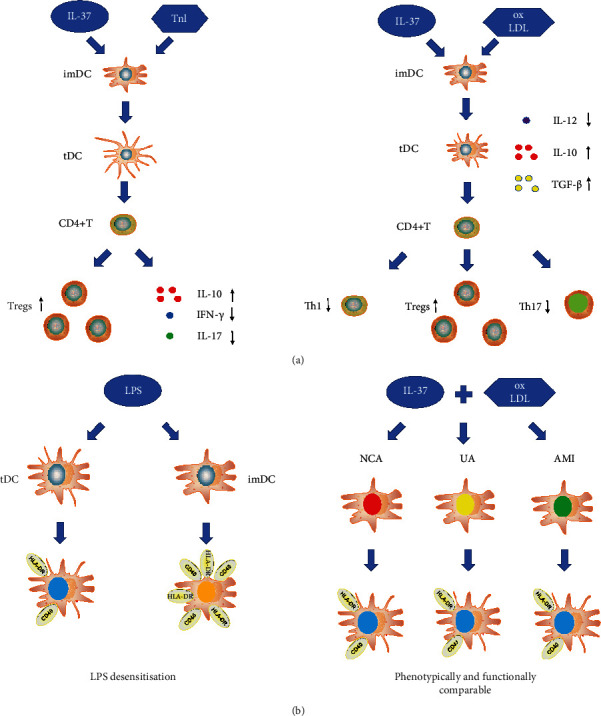
Ex vivo generation of tDCs and LPS desensitization. (a) DCs treated with IL-37 and TnI (oxLDL) display a tolerogenic phenotype. (b) LPS desensitization; IL-37-treated DCs from patients with ACS were phenotypically and functionally similar to IL-37-treated DCs from patients with NCA. TDCs: tolerogenic dendritic cells induced by troponin I (oxidized low-density lipoprotein) plus interleukin-37; LPS: lipopolysaccharide; IL-37: interleukin-37; TnI: troponin I; oxLDL: oxidized low-density lipoprotein; ACS: acute coronary syndrome; NCA: normal coronary artery.

**Figure 2:**
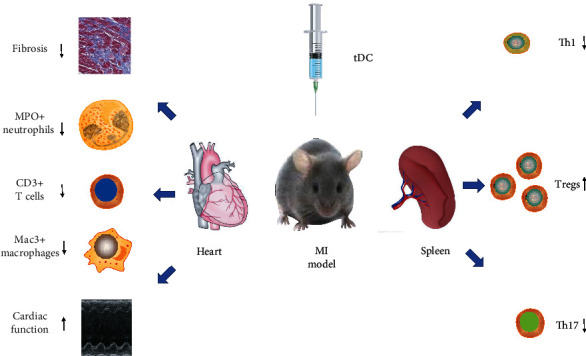
Protective role of tDCs in MI. In heart, adoptive transfer of tDCs inhibited cardiac fibrosis, decreased inflammatory cells infiltration, and prevented left ventricular function following MI. In spleen, adoptive transfer of tDCs increased the number of Tregs and decreased Th1 and Th17 cells. TDCs: tolerogenic dendritic cells induced by troponin I plus interleukin-37; MI: myocardial infarction; Tregs: regulatory T cells.

As emerging evidence points to the key role of DCs in tolerance, it is crucial to examine the role of DCs more actively in ACS. Previous investigations have demonstrated that upregulation of mature DCs in MI is associated with adverse left ventricular remodeling, and that DCs play a regulatory role in postinfarction healing and left ventricular remodeling in mice [86, 87]. Moreover, an observation from our laboratory showed that Kruppel-like factor 2 plays a regulatory role in DC activation in patients with ACS [88]. Apolipoprotein B100 (ApoB100), a composition of low-density lipoprotein (LDL), is a major factor leading to atherosclerosis. Of note, ApoB100 plus IL-10-treated DCs ameliorated atherosclerosis, while IL-37tg mice-derived bone-marrow-derived DCs displayed reduced costimulatory molecules and MHC-II after administration of LPS [11, 32, 89]. Furthermore, Liu et al. reported that IL-37 ameliorated the maturation of DCs in ApoE-/- mice [90]. Recently, our group revealed that IL-37 and Troponin I (TnI) treated DCs obtained a tolerogenic phenotype and alleviated cardiac remodeling after myocardial infarction [42] (Figures 1 and 2). The above two recent studies together suggested that IL-37 targets DCs and finally ameliorates atherosclerosis and cardiac remodeling after MI in mice. Interestingly, our immunohistochemical analysis showed that TnI-plus IL-37-induced tDCs ameliorated neutrophil, macrophage, and T-cell infiltration in post-MI hearts, whereas mature DCs deteriorated infiltration of these inflammatory cells [42]. These data imply that IL-37-induced tDCs inhibit the inflammatory response in infarcted hearts. In humans, we also showed that IL-37-treated DCs obtained the characteristics of tDCs, which could induce tolerance [9] (Figure 1(a)). Both our data and prior studies show that ventricular remodeling can be attenuated by limiting the acute inflammatory response [64, 91, 92]. Hence, we speculate that the suppression of inflammatory cell accumulation by adoptive transfer of IL-37 plus TnI–treated tDCs may represent a novel therapeutic strategy for ventricular remodeling after MI in the future.

Treatment with tDCs is just emerging, although the utility of immunogenic DCs for cancer therapy has evolved over 20 years in the clinical arena [21–23, 93, 94]. The first and very important obstacle for the utility of donor tDCs is that the immune system of the recipient may destroy these “foreign” cells [95]. However, autologous tDCs could resolve this issue. In our previous study, we observed that IL-37-treated DCs from patients with ACS were phenotypically and functionally similar to IL-37-treated DCs from normal coronary artery patients [9] (Figure 1(b)). The above important finding suggests the possibility of ex vivo generation of autologous tDCs for immunotherapy [28–30]. To the best of our knowledge, this is the first attempt to explore the probability of induction of tDCs in patients with ACS.

Another impediment to tDC therapy is whether the in vitro-induced tDCs are refractory to maturation when encountered with inflammatory stimuli in vivo. The conversion of tDCs into mature DCs aggravates disease severity [96], because immunogenic DCs play a major role in the initiation and regulation of adaptive immunity, but tDCs provide opportunities for therapy of autoimmune diseases [28, 97]. Inflammatory reactions are significantly inhibited following a second LPS stimulation after the first LPS challenge, which is termed as “LPS desensitization” [98–101]. As inflammation plays an important role in the development of ACS [1, 2], the in vitro-induced tDCs may convert into mature DCs when encountered with inflammatory stimuli in the context of ACS. Indeed, the tolerogenic properties of IL-37-treated DCs are very stable because the phenomenon of “LPS desensitization” was confirmed in these tDCs in our previous study [9] (Figure 1(b)). As oxidized low-density lipoprotein (oxLDL) plays an important role in the development of atherosclerosis and promotes mature dendritic cell transition [102], further studies are required to explore the shift in DCs treated with oxLDL and IL-37 exposed to a maturation stimulus (e.g., LPS or oxLDL) in a murine model of atherosclerosis. Collectively, these intriguing results suggest that self-IL-37-treated tDCs may provide a new treatment strategy for ACS patients in the future.

In summary, inflammation plays a central role in the development of ACS, and IL-37 is a natural inhibitor of innate and adaptive immunity. IL-37 significantly alleviated ventricular remodeling after MI and myocardial ischemia/reperfusion injury in mice. Although the priming of antigen-specific immune responses has been well studied, the function of DCs in immunologic tolerance is just beginning to be studied. IL-37 plus TnI-treated DCs were tolerogenic, and adoptive transfer of these antigen-loaded tDCs significantly increased the number of Treg cells and attenuated inflammatory cell infiltration. Importantly, we explored the role of IL-37 in ACS patients and the probability of induction of tDCs derived from ACS patients. Based on these intriguing findings, IL-37 or IL-37-treated tDCs may offer a new therapeutic target for ACS patients in the future. However, one limitation of the present review should be considered. Plasma IL-37 levels of ACS patients were measured by ELISA in several publications of this review, and the validation of IL-37 measurements by Western blot is needed to address this issue.

## Abbreviations

IL-37: Interleukin-37
ACS: Acute coronary syndrome
NCA: Normal coronary artery
LPS: Lipopolysaccharide
tDC: Tolerogenic dendritic cells
MI: Myocardial infarction
TnI: Troponin I
Tregs: Regulatory T cells
IFN-*γ*: Interferon*-γ*
TNF: Tumor necrosis factor
oxLDL: Oxidized low-density lipoprotein
ApoB100: Apolipoprotein B100
LDL: Low-density lipoprotein.

## Supplementary Materials

Supplementary Materials Figure S1 legend: protective role of IL-37 in MI. IL-37 significantly improved ventricular remodeling after MI. IL-37: Interleukin-37; MI: myocardial infarction.
