# Supplementary material for: Role of IL-37- and IL-37-Treated Dendritic Cells in Acute Coronary Syndrome
Source: Oxid Med Cell Longev. 2021 Aug 21;2021:6454177. doi: 10.1155/2021/6454177 (PMC8405329; doi:10.1155/2021/6454177)
Supplement: Supplementary Materials — Figure S1 legend: protective role of IL-37 in MI. IL-37 significantly improved ventricular remodeling after MI. IL-37: Interleukin-37; MI: myocardial infarction. [file 6454177.f1.pptx]

## Slide 1
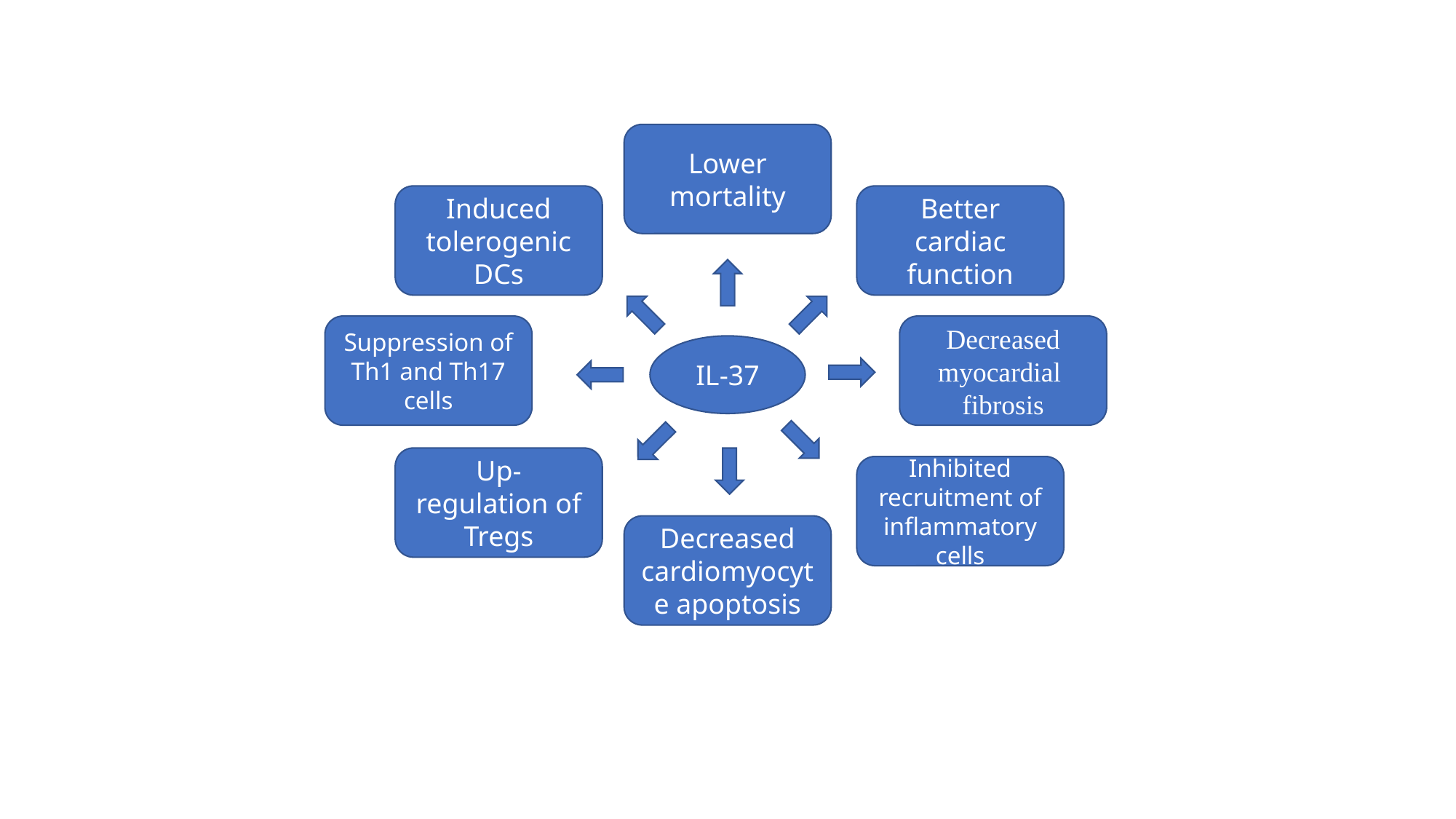

Lower mortality
Induced tolerogenic DCs
Better cardiac function
Suppression of Th1 and Th17 cells
Decreased myocardial
fibrosis
IL-37
Up-regulation of Tregs
Inhibited recruitment of inflammatory cells
Decreased cardiomyocyte apoptosis
